# Favorable Circulatory System Outcomes as Adjuvant Traditional Chinese Medicine (TCM) Treatment for Cerebrovascular Diseases in Taiwan

**DOI:** 10.1371/journal.pone.0086351

**Published:** 2014-01-27

**Authors:** Hsienhsueh Elley Chiu, Yu-Chiang Hong, Ku-Chou Chang, Chun-Chuan Shih, Jen-Wen Hung, Chia-Wei Liu, Teng-Yeow Tan, Chih-Cheng Huang

**Affiliations:** 1 Department of TCM, Chang Gung University College of Medicine, Kaohsiung, Taiwan; 2 Department of Neurology, Chang Gung University College of Medicine, Kaohsiung, Taiwan; 3 Department of Rehabilitation, Chang Gung University College of Medicine, Kaohsiung, Taiwan; 4 Chang Gung Memorial Hospital, Chang Gung University College of Medicine, Kaohsiung, Taiwan; 5 School of Chinese Medicine for Post-Baccalaureate, I-Shou University, Kaohsiung, Taiwan; St Michael's Hospital, University of Toronto, Canada

## Abstract

**Background:**

This study searches the National Health Insurance Research Database (NHIRD) used in a previous project, aiming for reconstructing possible cerebrovascular disease-related groups (DRG),and estimating the costs between cerebrovascular disease and related diseases.

**Methods and Materials:**

We conducted a nationwide retrospective cohort study in stroke inpatients, we examined the overall costs in 3 municipalities in Taiwan, by evaluating the possible costs of the expecting diagnosis related group (DRG) by using the international classification of diseases version-9 (ICD-9) system, and the overall analysis of the re-admission population that received traditional Chinese medicine (TCM) treatment and those who did not.

**Results:**

The trend demonstrated that the non-participant costs were consistent with the ICD-9 categories (430 to 437) because similarities existed between years 2006 to 2007. Among the TCM patients, a wide variation and additional costs were found compared to non-TCM patients during these 2 years. The average re-admission duration was significantly shorter for TCM patients, especially those initially diagnosed with ICD 434 during the first admission. In addition, TCM patients demonstrated more severe general symptoms, which incurred high conventional treatment costs, and could result in re-admission for numerous reasons. However, in Disease 7 of ICD-9 category, representing the circulatory system was most prevalent in non-TCM inpatients, which was the leading cause of re-admission.

**Conclusion:**

We concluded that favorable circulatory system outcomes were in adjuvant TCM treatment inpatients, there were less re-admission for circulatory system events and a two-third reduction of re-admission within ICD-9 code 430 to 437, compared to non-TCM ones. However, there were shorter re-admission duration other than circulatory system events by means of unfavorable baseline condition.

## Introduction

Cerebrovascular diseases pose a critical threat to human health. The prevalence of cerebrovascular diseases in the study population older than 45 years old was 17.5/1000 (95% confidence interval 17.0–18.0) [1]. Cerebrovascular diseases, such as hemorrhage, thrombosis and necrosis, are malignant and can cause death or disability, which has a detrimental effect on individuals, families, and society [2,3]. According to World Health Organization (WHO) reports, cerebrovascular diseases in high income countries were among the world's top 10 leading causes of death in 2005, and were the second highest cause of death overall. Every year, 770 000 people die from cerebrovascular diseases, and in middle-income countries they are the leading cause of death (3.14 million deaths per year). [4]. Taiwan’s Health Department reported that cerebrovascular disease has been among the top three leading causes of death for 10 consecutive years, and in 2005 and 2006, it was responsible for the second highest number of deaths (malignant tumors were the leading cause of death). Numerous inpatients survive with appropriate stroke management. However, acute stage patients are in a compromised situation that could easily lead to infections, it could result in a sequel of neurological deficits, such as limb, language, bowel control, vision, and emotional dysfunctions. The health burden of cerebrovascular diseases was measured at an annual population prevalence of 11.7/1000 (95% confidence interval of 11.3 to 12.1), and it is estimated that up to 67% of cerebrovascular disease survivors are unable to be self-supporting [1], and 10% of survivors require long-term care in paramedical institutions [5]. Cerebrovascular disease not only causes a great inconvenience for inpatients, but also has a tremendous impact on families. In addition, the cost to the community is high, and effectively managing the medical and social support costs remains a challenge.

Traditional Chinese medicine (TCM) has been used to treat cerebrovascular disease for millenary. Treatments include passive massage and Tai-Chi rehabilitation. Acupuncture and moxibustion have been used for at least two thousand years [6]. Traditional Chinese medicine acupuncturists select specific acupoints on both the healthy and affected side of the body, based on the Zang-Fu thesis for patients who clinically suffer hemiplegia. Most patients experience functional recovery to varying degrees. Numerous animal and human experiments have indicated that acupuncture can lead to improved blood circulation and induces various biochemical reactions. Acupuncture, moxibustion, and electro-acupuncture could cause localized biological reactions through remote response or systemic reactions. These treatments work mainly through the excitement of the central nervous pathway, and consequently influence more than one physical system, including the central and peripheral nervous systems [10–14]. Recently, acupuncture has become widely used around the world, and according to a statement by the National Institutes of Health in the U.S.A., the application of acupuncture has been recognized for cerebrovascular diseases [7,12]. Studies on acupuncture and moxibustion conducting in China and Europe indicated positive results for treating cerebrovascular disease [8]. A double-blinded study, using computed tomography, found that the severity of neuronal damage of patients who accepted acupuncture treatment was half that of patients who did not accept. In addition, acupuncture reduced the severity of motor paralysis [9].

Since 2006, the Pilot Scheme of National Health Policy in Stroke Adjuvant Acupuncture and Traditional Chinese Medicine (TCM) Therapy has been applied. Our previous reports demonstrated that verum acupuncture, in moderate to severe ischemic stroke patients, might play a protective role against comorbidity and mortality during admission, as well as for 6 months after discharge. However, the studies did not find improvements in neurological deficits [15]. Thus, the severe stroke burden contributed to clinical and economic policies. We therefore conducted a nationwide retrospective cohort analysis in stroke inpatients, aiming for reconstructing possible cerebrovascular disease-related groups (DRG),and estimating the costs between cerebrovascular disease and related diseases. we examined the overall costs in three municipals in Taiwan, and estimated the possible costs for the expecting diagnosis related group (DRG) using the ICD-9 system, and an overall analysis of the re-admission population (including inpatients who did and did not receive TCM treatment).

## Methods and Materials

We analyzed inpatient costs using the 2006 to 2007 research database. Ambulatory care costs were analyzed using the profiles international classification of diseases version-9 (ICD-9) code 430 to 437 from the research database of the National Health Insurance (NHIRD) to determine attributes such as in-patient duration-time, age, and sex. The investigation project was approved by the ethical committee of Chang Gung Memorial Hospital (Institution Review Board No.99-1800B). The apriori association rule was used to perform data mining using the primary ICD-9 code to identify cerebrovascular disease-related groups. To analyze expenses, we first combined the identity number (ID) and the first date of admission (IN_DATE) column as an index key to distinguish duplicative data based on a screen of the ICD code (430–437). Second, we classified medical institution types based on column of contract hospital type (HOSP_CONT_TYPE). Third, if the department type for selecting if Chinese Medical Treatment records (FUNC_TYPE) column number 60–69 existed, we considered it as a TCM treatment. In Taiwan, acupuncture and Chinese Traumatology manipulation are classified as one TCM therapeutic method. Finally, to determine whether combined TCM and western medical treatment data existed, we used the ID column to compare the medical records.

**Figure 1:**
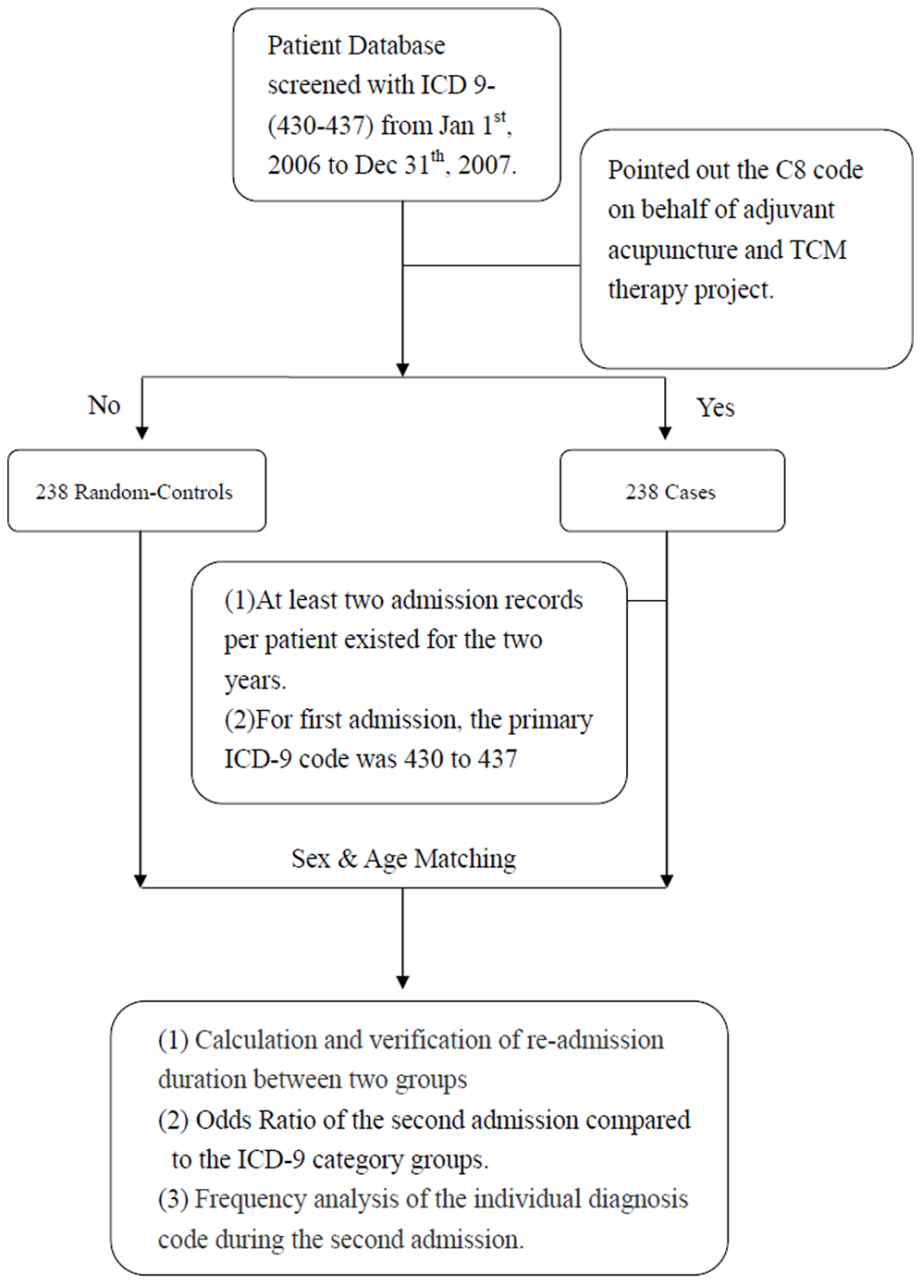
Analysis Strategy (2006–2007).

Because the detail document (DD document) of the hospital medical costs database is screened under the main diagnosis code bar unit and filtered under a cerebrovascular code from the research database of the Bureau of National Health Insurance, the first time follow-up patient data were analyzed by (1) merging the ID digital number field and the hospital date as the index key, which was differentiated using repeat gender data, (2) distinguishing between medical institution and special category field outpatient data, (3) regarding the column of interview specialty on medical character, if a TCM code (60 to 69) appeared, we defined it as a rational to distinguish between TCM and western medicine patient treatments, (4) finally, comparing ID digital numbers to identify if any duplications existed in the combination of TCM and western medicine treatments. The analysis of outpatient costs included outpatient visits, and character counting, and was scaled to 100,000 inpatients and the details of the drug and diagnosis and treatments, and pharmacy services costs, as well as information on the amount and average amount of costs related to partial cash payments for treatments. A decomposition analysis of medical cost was conducted to investigate cost trends compared to average medical costs per annum per person. All medical causes of the disease were influenced by age. Therefore, high costs are an accepted factor for aging populations, and result in a rise in total costs. In addition, the average age of the population is growing, which influences average costs. Therefore, the first step of the medical treatment cost analysis was to consider the average age of the population and include an analysis of the population structure. We filtered inpatients out using the ICD-9 code (430 to 437), and included all inpatients that received TCM treatments (case group), and also evaluated the re-admission inpatients database for the 2 years covered by this study. The random-control population was selected from inpatients who did not receive TCM treatments.(Figure 1) The multivariate Cox proportional hazard models by adjusting age, sex, occupation, low income, diabetes, hypertension, hyperlipidemia, mental disorder, anticoagulant medication, anti-platelet medication, and lipid-lowering medication. The odds ratio was calculated by Chi-square test according to 17 disease categories. The 95% confidence interval is analyzed for the significance of OR. Independent T test is used for continuous variable such as age variable. If the sample size violated parametric assumption, we replaced Independent T test with Mann-Whitney U test. Defied as statistical significance if p value is less than 0.05. The impact of adjuvant TCM therapy on the frequency and expenditure of emergency care and hospitalization after the index stroke admission, were analyzed in the general linear regressions by adjusted for age, sex, occupation, low income, coexisting medical conditions, stroke-related medication, medical center, types of stroke, history of stroke, length of stay, and neurosurgery.

## Results

In 2006, the inpatients’ first ICD-9 code between 430–437 as the primary analysis population, random-control group matched for sex, age by propensity score matching procedures. (Table 1) The adjusted hazard ratios (HRs) and 95% confidence intervals (CIs) of post-stroke complications and mortality associated with adjuvant TCM treatments were calculated again in ICD-9 codes. The number of hospitalizations in 2006–2007 was of 3,140,164 people, while the random-control group was 131,789 inpatients (deduction of TCM consultation inpatients within ICD-9 code 430–437). Adjuvant TCM treatment inpatients as the first diagnosis code within 430–437 were 558 inpatients, which including 238 re-admission ones.

**Table 1:** Database of Case Groups.

ICD-9 CM_CODE	Number of Cases (First admission)	Number of Re-admissions	Duration (days)	Age
430	40	20	55.90±60.66	52.85±18.49
431	209	93	67.58±86.15	60.56±12.68
432	0	0	-	-
433	8	6	128.50±109.84	66.83±9.93
434	291	114	67.00±88.55	66.46±12.01
435	1	1	30	58
436	3	1	5	74
437	4	3	58.33±60.75	67.33±2.89

Analysis showed that the adjuvant TCM treatment group had less odds (68.4%/31.6% = 2.16) than non-TCM treatment group (87.4%/12.6% = 6.93), favorable odds on non-TCM treatment group by re-admission ICD-9 code 430 ∼ 437 (relative risk ratio, 6.93/2.16 = 3.2), 95% confidence interval not containing 1, P<0.01. A two-third reduction of re-admission within ICD-9 code 430 ∼ 437 favored the adjuvant TCM treatment group, the difference was statistically significant.

**Figure 2:**
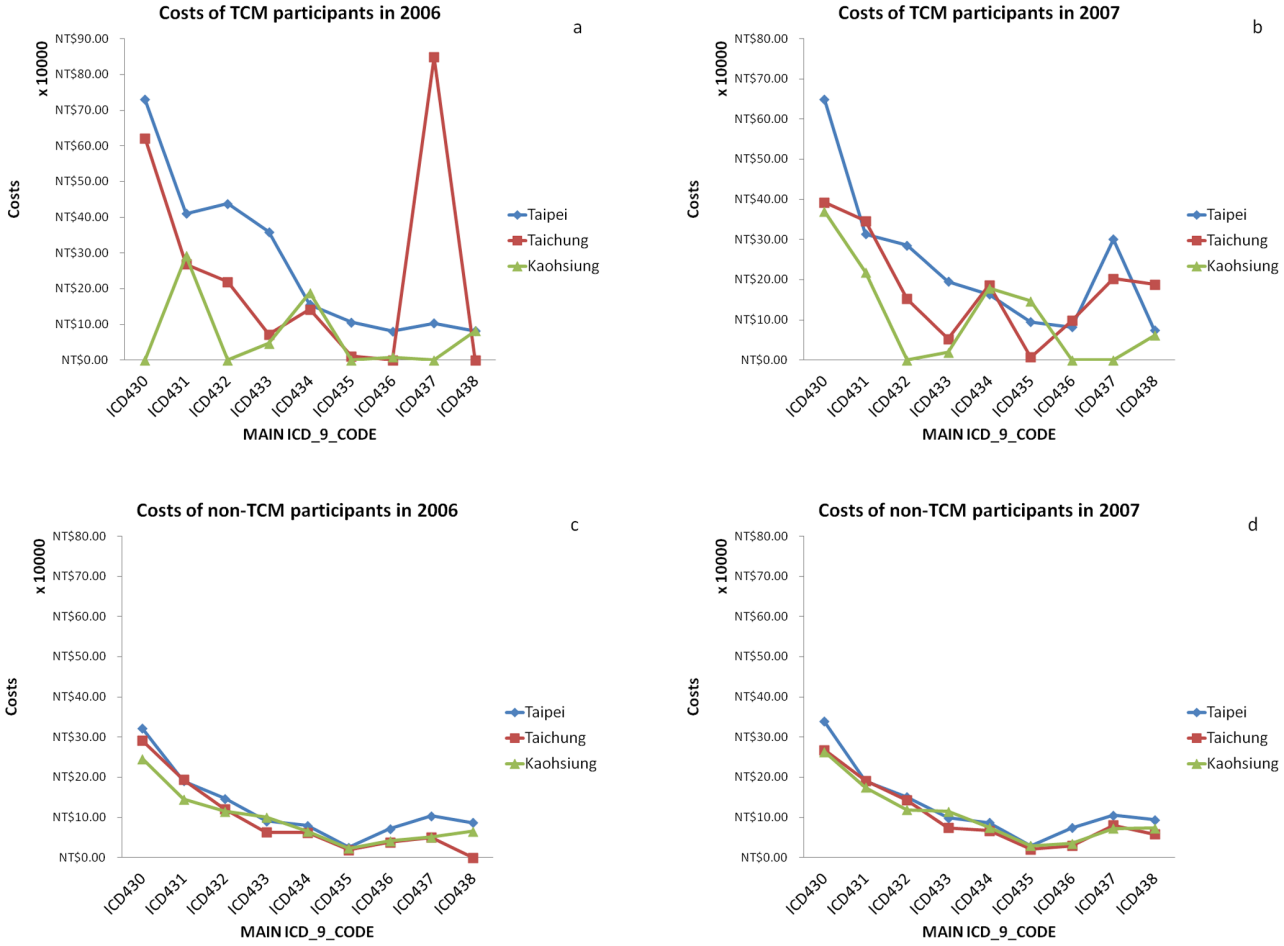
The inpatients expenditures as admission in year 2006 and 2007 (by insurance covered city).

We estimated that overall, only 4% of the annual budget was spent on TCM treatments, and the rest was spent on conventional treatments. We investigated three municipals— Kaohsiung, Taichung and Taipei, they are on behalf of the south, the middle and the north part of Taiwan. The medical expenditure trend indicated that the costs for non-TCM treatment inpatients were consistent in the ICD-9 category (430 to 437), and were similar for both years. However, wide variation and more costs were incurred by TCM participants than non-TCM ones during these 2 years.(Figure 2)

**Figure 3:**
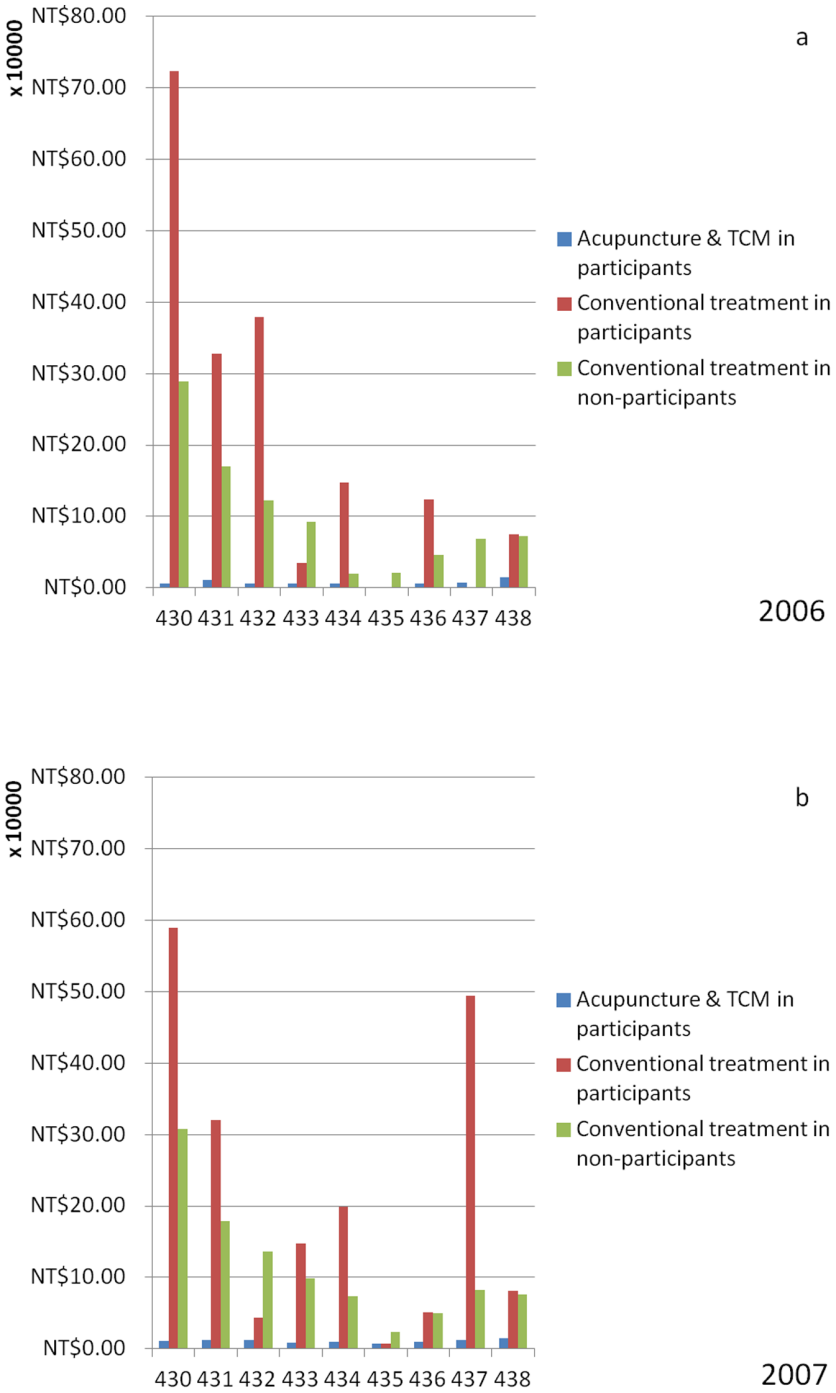
The inpatient expenditures as admissions in each diagnosis related groups (DRG) in year 2006 (3a) and 2007 (3b).

The overall secondary costs to western medicine treatment for all inpatients were more in 2006, as indicated by ICD 430, 432, 434, and 436, than in 2007. In 2007, ICD 433 and 437 reported higher costs than in 2006. In 2007, the trend demonstrated that adjuvant TCM treatment inpatients paid less for conventional treatments, with the exception of ICD 437, which appeared to be higher in the first instance.(Figure 3)

**Figure 4:**
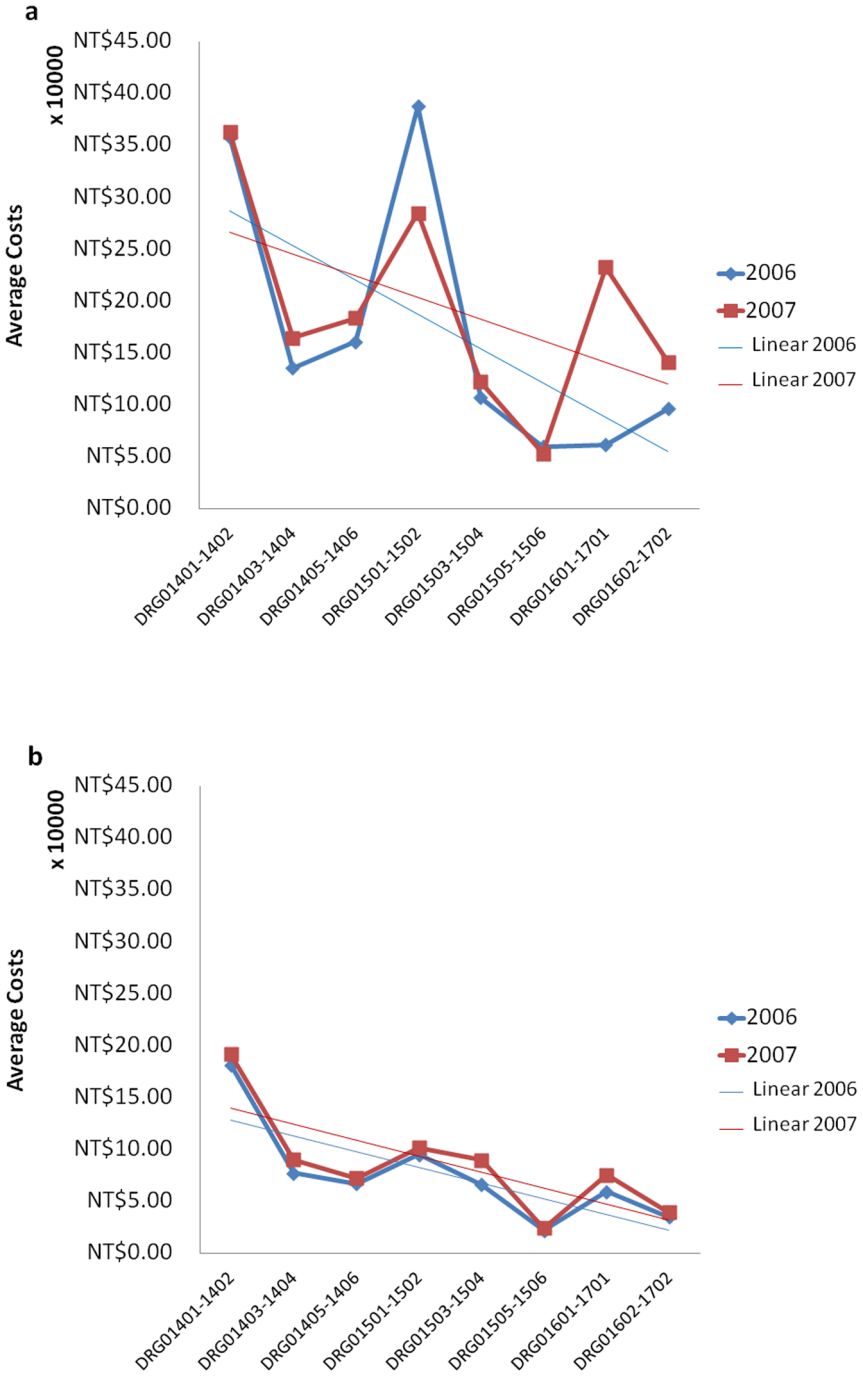
Expected costs of TCM participants (4a) and non-TCM participants (4b) with DRG in year 2006 and 2007.

This study demonstrated that for inpatients receiving TCM treatments for the DRG1501-02 Project, the annual costs during 2007 were significantly lower than in 2006. By contrast, for DRG1601-1702, if the TCM associated medical costs were excluded, the medical treatment costs for the 2 years were similar.(Figure 4)

**Figure 5:**
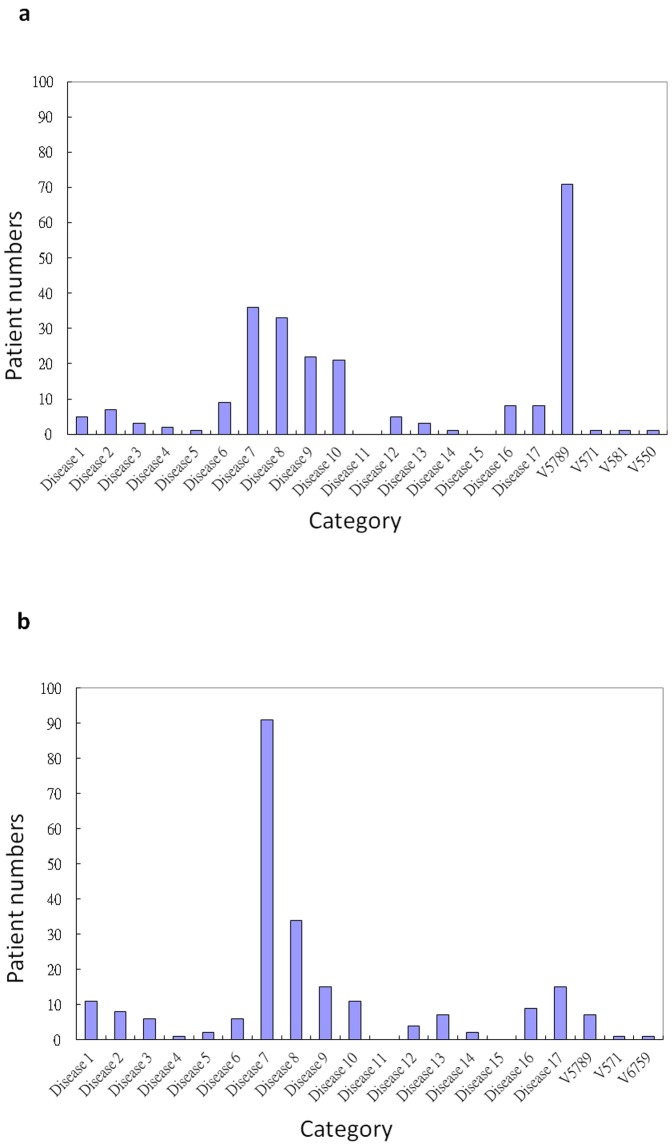
The distribution of ICD-9 category in re-admission of cases (5a) and random-controls (5b) group.

During the first hospitalization, the percentage of adjuvant TCM treatments was 0.82% in 2006 and 1.70% in 2007. In addition, the re-admission rate of TCM inpatients was 17.84% in 2006 and 19.04% in 2007. During the first hospitalization, 99.18% (2006) and 98.3% (2007) of the whole population were non-TCM inpatients, and the re-admission rate was 10.56% in 2006 and 9.43% in 2007. The average re-admission duration was significantly shorter for TCM participants, especially for the initial ICD 434 cases.(Table 2) However, during subsequent re-admission episodes, the odds ratio for Disease 7 of ICD-9 category, representing circulatory system diseases, significantly favored non-TCM participants among all potential causes. (Table 3)(Figure 5)

**Table 2:** Duration of Re-admission Cases and Random-Controls.

ICD-9 CM_CODE	Numbers of Re-admissions	Sex	Age	Control Population	Re-admission Duration (days)		*P*
					Cases	Controls	
430	20	M: 12	44.17±16.19	3820	66.58±76.09	55.83±61.79	.707
		F: 8	65.88±13.88		39.88±19.99	115.38±173.07	.258
431	93	M: 63	58.32±12.90	21281	63.40±76.77	89.35±142.98	.219
		F: 30	65.27±10.96		76.37±104.04	127.67±170.26	.165
433	6	M: 4	62.00±7.12	4664	143.75±127.23	283.50±232.20	.331
		F: 2	76.50±7.78		98.00±94.75	103.50±126.57	.965
434	114	M:59	62.56±12.38	73784	63.00±78.05	134.21±149.94	.001
		F: 55	70.65±10.14		71.29±99.14	115.77±129.83	.047
435	1	M:1	58	17852	30	167	-
		F: 0	-		-		-
436	1	M: 0	-	6203	-		-
		F: 1	74		5	381	-
437	3	M:1	69	4185	1	222	
		F: 2	66.50±3.54		87.00±49.49	198.00±142.84	.408

**Table 3:** Odds Ratio of Major Disease Re-admission in Cases*/* Random –Controls.

Disease Group	OR			Lower 95% CI			Upper 95% CI		
	total	*men*	*women*	total	*men*	*women*	total	*men*	*women*
Disease 1	0.43	0.40	0.48	0.12	0.07	0.04	1.37	1.81	3.45
Disease 2	0.84	1.97	0.19	0.26	0.41	<0.01	2.72	12.39	1.74
Disease 3	0.48	0.96	-	0.08	0.13	-	2.28	7.32	-
Disease 4	1.95	-	-	0.10	-	-	115.49	-	-
Disease 5	0.48	0.96	-	0.01	0.01	-	9.35	76.23	-
Disease 6	1.47	1.72	0.97	0.46	0.43	0.07	5.11	8.21	13.77
Disease 7	0.27*	0.20*	0.44*	0.17	0.10	0.21	0.44	0.37	0.91
Disease 8	0.93	1.07	0.75	0.54	0.52	0.30	1.62	2.24	1.85
Disease 9	1.47	1.17	2.07	0.70	0.45	0.61	3.13	3.15	8.00
Disease 10	1.94	2.00	1.90	0.87	0.60	0.61	4.55	7.65	6.51
Disease 11	-	-	-	-	-	-	-	-	-
Disease 12	1.22	0.48	2.00	0.26	0.01	0.28	6.22	9.31	22.53
Disease 13	0.41	0.64	0.24	0.07	0.05	<0.01	1.82	5.67	2.47
Disease 14	0.48	-	-	0.01	-	-	9.35	-	-
Disease 15	-	-	-	-	-	-	-	-	-
Disease 16	0.86	1.21	0.57	0.28	0.25	0.09	2.56	6.25	3.06
Disease 17	0.50	0.31	0.63	0.18	0.03	0.17	1.29	1.79	2.08

Disease group means ICD-9 category 1 to 17;

OR means odds ratio.

* means *P* <.01, favors random-controls.

## Discussion

Among inpatients who were diagnosed with ICD-9 code (430 to 437) upon first admission, the average re-admission duration was significantly shorter for TCM inpatients. In addition, the inpatients being introduced to TCM demonstrated more severe general symptoms, which were costly to deal with conventional treatments, and the severity of underline diseases might be causative re-admission. However, adjuvant TCM treatment inpatients appeared to have significantly lower circulatory system events while re-admission. However, there were shorter re-admission duration other than circulatory system events by means of unfavorable baseline condition.

The disease diagnosis related group (DRG) measure enforces equal medical fee payments for diagnosis-related groups and helps to reduce unnecessary medical costs that burden the system. Recently, Taiwan has been planning to implement the DRG. However, after numerous meetings, consultations, commissioning of scholars and experts to lead relevant research projects, some public interest-related support measures have been developed for the short-term, but no guarantee exists that public health interests are not negatively influenced by the waste of additional medical resources. Since 2009, DRG measures have focused on the gradual implementation of health payments with a pilot project. This study’s design was based on a previous study focusing on data from 2006 and 2007 Taiwanese National Health Insurance Research Database, and included study participants that were diagnosed under the international standard code for diagnosis of disease (ICD-9) in the cerebrovascular disease Group (430 to 437) for analysis. In April 2006, the National Health Council issued a Pilot Scheme of Health Policy in Stroke Adjuvant Acupuncture and TCM Therapy [15], and we used this scheme to investigate the statistics on TCM participation per patient per year for all inpatients hospitalized during 2006 and 2007. We then analyzed the overlapping use of integrative Chinese and Western medicine in hospitals to understand regional differences between inpatients that participated in pilot scheme in three municipals by analyzing the costs of admission and number of outpatients. In addition, we partially launched the DRG project in 2011. The 2006 to 2007 database of the NHIRD was used to perform cost predictions for retrospective classifications. However, the national medical institutions failed to timeously implement the pilot scheme in 2006, and ragged data could not be avoided. Therefore, additional research of the health insurance database is needed.

## Conclusion

Among inpatients diagnosed with ICD-9 code 430 to 437 upon first admission, the adjuvant TCM treatment patients appeared to be significantly less re-admission for circulatory system events. A two-third reduction of re-admission within ICD-9 code 430 ∼ 437 favored the adjuvant TCM treatment patients.

## Supporting Information

File S1 **Appendix S1.**
(DOCX)
