# Correction: Favorable Circulatory System Outcomes as Adjuvant Traditional Chinese Medicine (TCM) Treatment for Cerebrovascular Diseases in Taiwan

**DOI:** 10.1371/journal.pone.0091603

**Published:** 2014-02-28

**Authors:** The PLOS ONE staff

The name of the second author is spelled incorrectly. The correct name is: Yu-Chiang Hung.

The correct Citation is: Chiu HE, Hung Y-C, Chang K-C, Shih C-C, Hung J-W, et al. (2014) Favorable Circulatory System Outcomes as Adjuvant Traditional Chinese Medicine (TCM) Treatment for Cerebrovascular Diseases in Taiwan. PLoS ONE 9(1): e86351. doi:10.1371/journal.pone.0086351.
