# Supplementary material for: Favorable Circulatory System Outcomes as Adjuvant Traditional Chinese Medicine (TCM) Treatment for Cerebrovascular Diseases in Taiwan
Source: PLoS One. 2014 Jan 27;9(1):e86351. doi: 10.1371/journal.pone.0086351 (PMC3903523; doi:10.1371/journal.pone.0086351)
Supplement: File S1 — Appendix S1. (DOCX) [file pone.0086351.s001.docx]

Appendix

1. Abbreviations

HOSP_CONT_TYPE- Contract Hospital Type

| Code | Name |
| --- | --- |
| 1 | Academic Medical Centers |
| 2 | Metropolitan Hospitals |
| 3 | Local Community Hospitals |
| 4 | Physician Clinics & Dental Clinics |

IN_DATE- the first date of admission in YYYYMMDD Format

FUNC_TYPE-Department type for selecting if there exist Chinese medical treatment records.

1. The Index of seventeen diseases

| Disease Category | Disease Name | ICD_CODE range |
| --- | --- | --- |
| 1 | INFECTIOUS AND PARASITIC DISEASES | 001-139 |
| 2 | NEOPLASMS | 140-239 |
| 3. | ENDOCRINE, NUTRITIONAL AND METABOLIC DISEASES, AND IMMUNITY DISORDERS | 240-279 |
| 4. | DISEASES OF THE BLOOD AND BLOOD-FORMING ORGANS | 280-289 |
| 5. | MENTAL DISORDERS | 290-319 |
| 6. | DISEASES OF THE NERVOUS SYSTEM AND SENSE ORGANS | 320-389 |
| 7. | DISEASES OF THE CIRCULATORY SYSTEM | 390-459 |
| 8. | DISEASES OF THE RESPIRATORY SYSTEM | 460-519 |
| 9. | DISEASES OF THE DIGESTIVE SYSTEM | 520-579 |
| 10. | DISEASES OF THE GENITOURINARY SYSTEM | 580-629 |
| 11 | COMPLICATIONS OF PREGNANCY, CHILDBIRTH, AND THE PUERPERIUM | 630-677 |
| 12. | DISEASES OF THE SKIN AND SUBCUTANEOUS TISSUE | 680-709 |
| 13. | DISEASES OF THE MUSCULOSKELETAL SYSTEM AND CONNECTIVE TISSUE | 710-739 |
| 14. | CONGENITAL ANOMALIES | 740-759 |
| 15. | CERTAIN CONDITIONS ORIGINATING IN THE PERINATAL PERIOD | 760-779 |
| 16. | SYMPTOMS, SIGNS, AND ILL-DEFINED CONDITIONS | 780-799 |
| 17. | INJURY AND POISONING | 800-999 |
